# Cardiovascular Intervention in Neonates Using an Umbilical Vein Approach

**DOI:** 10.3390/children8111017

**Published:** 2021-11-05

**Authors:** Ying-Tzu Ju, Yu-Jen Wei, Yung-Chieh Lin, Min-Ling Hsieh, Jing-Ming Wu, Jieh-Neng Wang

**Affiliations:** 1 Department of Pediatrics, National Cheng Kung University Hospital, College of Medicine, National Cheng-Kung University, Tainan 704302, Taiwan; ints.ju@gmail.com (Y.-T.J.); garlicwei@hotmail.com (Y.-J.W.); appledr@hotmail.com (Y.-C.L.); mactep_8@hotmail.com (M.-L.H.); jingming@mail.ncku.edu.tw (J.-M.W.); 2 Institute of Clinical Medicine, College of Medicine, National Cheng-Kung University, Tainan 701401, Taiwan; 3 Department of Pediatrics, College of Medicine, National Cheng-Kung University, Tainan 701401, Taiwan

**Keywords:** umbilical vein, newborn, premature infants, cardiovascular intervention, patent ductus arteriosus

## Abstract

Cardiovascular catheterization has been applied in infant treatment for several decades. To date, considerable research attention has been paid to cardiovascular catheterization in small neonates. However, peripheral vascular routes of catheterization are possible obstacles for interventionists. Umbilical vein catheterization has been reported as a route for neonates, although few attempts have been made to investigate this approach. This study aimed to retrospectively review cardiovascular intervention using the umbilical vein approach as applied to infants admitted to a tertiary center from 2017 to 2020. Details including the perinatal variables, indication diagnoses, and procedure devices were collected. The enrollment included a total of 16 cases representing 17 intervention events, with infants born at a gestation age of 22–39 weeks and body weight ranging from 478 to 3685 g at the time of the procedure. The postnatal age ranged from 1 to 27 days. The catheter sizes ranged from 4 to 11 Fr. Indications included being admitted for patent ductus arteriosus occlusion (*n* = 15), balloon pulmonary valvuloplasty (*n* = 3), balloon atrial septostomy (BAS) (*n* = 3), pulmonary valve (PV) perforation (*n* = 1), and two interventions for catheter placement for continuous venovenous hemofiltration. The success rate for cardiovascular catheterization was 88.2% (15/17). There were two patients for which cannulation failed due to ductus venosus closure: one intraabdominal hemorrhage complication during continuous venovenous hemofiltration (CVVH), and one cardiac catheterization failure of PV perforation due to failure to insert the guiding catheter into the right ventricular outflow tract. Based on these findings, we conclude that cardiac catheterization and the placement of a large-sized catheter through an umbilical vein in a small infant represents a safe and time-saving method when catheterization is required.

## 1. Introduction

Advances in the medical and surgical management of neonates are often predicated upon secure vascular access [1]. Recent advances in interventional cardiovascular procedures applied during the neonatal period are alternative life-saving methods used both for the urgent correction of clinical conditions and initial palliative therapy prior to surgery [2,3]. The percutaneous approach to central venous cannulation is sometimes difficult in neonates, especially in premature infants. Most commonly, the femoral vein, or femoral artery, is used for vascular access in the cardiac catheterization of children. Even with ultrasound guidance, setting up vascular access in infants remains challenging, especially in premature infants with a birth body weight of less than 1000 g [4,5]. By contrast, umbilical vein catheterization is a common and relatively quick and easy procedure in the neonatal intensive care unit (NICU) care [6]. The use of the umbilical vessel in cardiac catheterization was first reported in 1961 for angiography, but has rarely been mentioned of late due to recent advances in cannulation techniques [7]. However, the increasing frequency of even smaller and lower body weight premature neonates is resulting in an increased demand for less invasive cardiovascular interventions and central venous cannulation. Therefore, an umbilical vein approach is a possible alternative to cannulation [8]. Here, we report our experiences of using the umbilical vein as a route for intervention.

## 2. Materials and Methods

### 2.1. Study Design

This study recruited patients admitted to the NICU in our institution from 1 January 2017 to 31 December 2020. Those with an age of less than 1 month and with umbilical venous catheter insertion for a double lumen catheter or for cardiac catheterization were included. Those who did not receive umbilical vein catheter insertion were excluded. Infants with an umbilical venous catheter used only for intravenous drug or parenteral nutrition were also excluded. Infants with congenital anomaly, except for isolated congenital heart disease, were excluded. Infants with numerical or mosaic chromosomal anomaly, diagnosed prenatally or after birth, were also excluded.

Infants with umbilical vein access used for cardiac catheterization were further divided into successful intervention (Group A) and failed intervention (Group B) groups. The study protocol was approved by our Institutional Review Board (protocol code: A-ER-110-197).

### 2.2. Study Setting

This study was conducted in a 20-bed tertiary NICU at the National Cheng Kung University Hospital in Tainan, Taiwan. The care volume of this unit is approximately 350–400 neonates treated yearly. Admitted infants are regularly cared for by two neonatologists, two residents, and one nurse practitioner.

### 2.3. Study Setting Procedure

**Figure 1:**
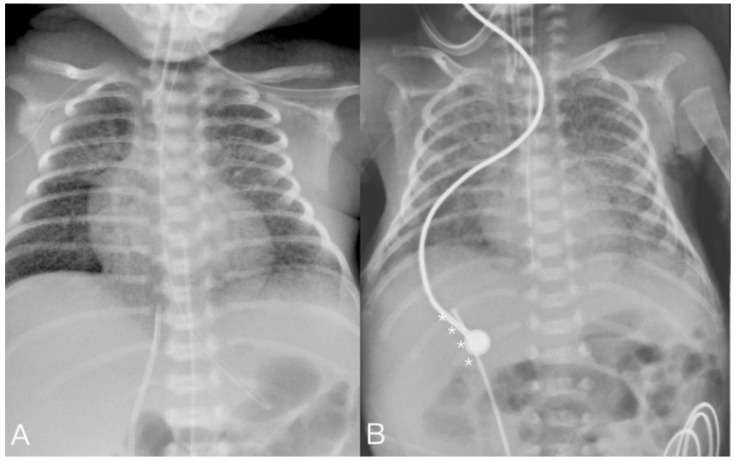
(**A**) AP view of correct UV catheter position. The catheter tip went straight up or slightly leftward to the diaphragm (Case 8). (**B**) AP view of incorrect UV catheter position. The catheter tip went rightward (asterisk) and not into the DV (Case 5).

**Figure 2:**
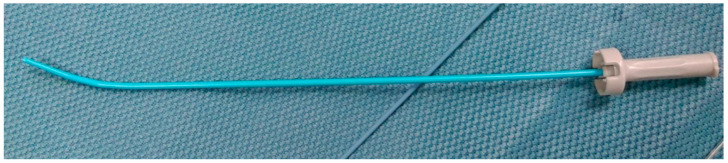
Introducer sheath dilator tip (Terumo 5 Fr.) was slightly bent manually. The preshaped introducer dilator could help the sheath pass more easily through the angle of ductus venosus and IVC.

The umbilical vein (UV) catheter was set up at the NICU by the pediatric resident doctor as standard umbilical catheterization. A 4 Fr. two-lumen central venous catheter (CVC) (Arrow ^®^ International, Inc., Cleveland, OH, USA) or a 3 Fr. one-lumen umbilical vein catheter (Vygon, Ecouen, France) was directly and smoothly inserted through the umbilical vein and then withdrawn to confirm that the catheter was in the vascular lumen. After catheter placement, the tip position was observed using a plain chest X-ray. The ideal position of the UV catheter tip is at the junction of the inferior vena cava (IVC) and right atrium [9,10,11]. In cases of correct placement, the catheter showed a slight curvature to the right of the midsagittal line between T10 to L1 before entering the ductus venosus, and then continued straight up or slightly leftward with the tip placed near the diaphragm level (about T8–T10 level) or in the right atrium [10,12]. In cases of incorrect placement, the catheter tip went rightward, and was not placed in the ductus venosus (DV) (Figure 1). In this situation, we addressed the incorrectly positioned catheter in the catheterization lab. A 0.014-inch Runthrough guidewire was inserted into the catheter, followed by retrieving the catheter to the level of the umbilical vein. We then advanced the guidewire through the ductus venosus to the IVC under fluoroscopy guidance. In all cases, the procedure was conducted at the catheterization laboratory, except in one case which included setting up the route for continuous venovenous hemodialysis (CVVH) at the NICU and the umbilical vein catheter being inserted directly. At the catheterization laboratory, the umbilical vein catheter was sterilized and cut. A 0.018-inch guidewire was used to change the catheter to a 4 Fr. introducer sheath (Merit Prelude, 7 cm) (Merit Medical Systems Inc., South Jordan, UT, USA). We then either used the sheath or appropriately altered its size (5 Fr. (Terumo sheath 7 cm) (Terumo Medical Corporation, MD, USA), 6 Fr. (Merit Prelude sheath, 7 cm) (Merit Medical Systems Inc., South Jordan, UT, USA), or 11 Fr. (Flexxicon dialysis catheter, 12.5 cm) (Bard Access Systems Inc., Salt Lake City, UT, USA)) for the procedure under fluoroscopy. In the catheterization laboratory, we changed the umbilical vein catheter to the introducer sheath under fluoroscope. The sheath dilator was straight and slightly stiffer than the umbilical vein catheter. To pass through the angle between the ductus venosus and IVC more smoothly, we slightly bent the introducer sheath dilator tip manually. The preshaped introducer sheath dilator could help the sheath pass more easily through the angle of the ductus venosus and IVC (Figure 2). We also attempted to lightly press the patient’s subxiphoid area, which could help the catheter pass through [13]. Changing the catheter with a guidewire also prevented transient constriction of the ductus venosus [14]. If the wire did not pass smoothly through the catheter until the IVC, injection of a small amount of contrast agent could reveal the patency of the ductus venosus (Supplementary Video S1). Finally, the sheath tip was placed at the junction of the IVC and right atrium to prevent ductus venosus spasm.

### 2.4. Clinical Variables

The basic demographic data comprising gestational age, birth body weight, indications for umbilical vein catheterization, type and size of catheter or sheath used, procedure type and equipment, age at time of procedure, and body weight were collected. Fluoroscopy and procedure time, complications, adverse events, and intervention outcome were also recorded.

## 3. Results

### 3.1. Enrollment of Study Population and Clinical Variables

During the study period, a total of 47 neonates received cardiac catheterization and 1 other neonate received CVVH through the umbilical vein. Among these infants, 32 were cannulated using femoral routes (3 from the subclavian artery, 2 from the femoral artery, 20 from the femoral vein, and 7 from the femoral artery and vein) and were excluded. The remaining 16 infants were included for analysis, and 15 of them received cardiac catheterization (Table 1). A total of 10 were premature infants. The birth body weight (BBW) was 1869 ± 1088 g (462 to 3705 g), and the birth gestational age (GA) was 32.5 ± 6.5 weeks (22 to 39 weeks). The age and body weight on the day of the procedure were 6.1 ± 6.3 days (1 to 27 days) and 1894 ± 1099 g (478 to 3685 g), respectively. The maximal cannulation catheter size was 11 Fr. in a 2356 g infant (Case 4). The patient had hemodynamically significant PDA and bilateral renal hypoplasia diagnosed after birth. After PDA occlusion, the umbilical vein was used for CVVH. Hence, a total of 17 interventional events (15 cardiac catheterization and 2 CVVH) in 16 infants were included in the analysis.

### 3.2. Intervention Details

The 15 cardiac catheterization events included 8 PDA occlusions (4 presented with pulmonary hemorrhage) (47.1%), 3 balloon pulmonary valvuloplasty (17.6%), 3 balloon atrial septostomy (BAS) (17.6%), and 1 pulmonary valve (PV) perforation (5.9%) (Table 2). The procedure time was 58.0 ± 38.9 min, and most cases were completed within 1 h (10/15). The fluoroscopy time was 15.1 ± 14.2 min.

There were 2 cases of umbilical vein cannulation failure (Case 5 and 13) that were due to a closed ductus venous (DV), and the wire failed to pass into the inferior vena cava. The remaining 13 cardiac catheterizations were performed successfully. However, there were 2 unsuccessful cardiac catheterization cases. In Case 9, the PDA was initially successfully closed using a UV approach, but hemodynamic instability occurred, although echocardiography did not reveal cardiac structure or pulmonary or systemic artery compression related to the device. Due to the unstable hemodynamic condition, we immediately retrieved the device. In Case 16, the guiding catheter could not advance into the right ventricular outflow tract from the UV route. The procedure was shifted to surgical pulmonary valvotomy due to failure to achieve cannulation in femoral vessels.

In total, 2 infants received CVVH therapy through the umbilical vein. Case 4 was an infant with bilateral renal hypoplasia. The infant was initially under CVVH therapy through the jugular venous route, but the cannulation site kept bleeding, and the catheter was dysfunctional. After PDA occlusion from UV for significant left-to-right shunting and heart failure, we cannulated the umbilical vein with an 11 Fr. double-lumen catheter for CVVH. During the CVVH course, abdominal distention with mild intraabdominal hemorrhage was found, which we considered to be related to a large sheath. The clinical condition stabilized after blood transfusion. Case 6 was an infant with acute kidney injury after birth and received CVVH through an 8 Fr. double-lumen catheter cannulated in the UV. The etiology was not discovered, and his renal function recovered spontaneously after 14 days of renal replacement therapy.

The total UV cannulation success rate was 88.2% (15/17), and the overall success rate of the procedure was 76.5% (13/17). The complication rate was 5.9% (1/17).

## 4. Discussion

The percutaneous approach to central venous catheterization is sometimes difficult in neonates, especially in premature infants. The National Institute for Health and Care Excellence (NICE) guidelines specify mandatory real-time 2D ultrasound in cases of percutaneous puncture of the internal jugular and femoral veins [15]. Even with ultrasound guidance, setting up vascular access in infants remains challenging, especially in premature infants with a birth body weight of less than 1000 g [4,5]. Umbilical vein catheterization is a common procedure in NICUs. The procedure was first reported in 1946 as a route for exchange transfusion [16]. The umbilical vein offers a technically easy, relatively safe and pain-free portal for intravascular catheter access in newborns. An umbilical vein catheter (UVC) provides a good alternative to a peripheral venous catheter by reducing the need for multiple procedures to maintain venous access, while not being associated with the greater risks of infection or necrotizing enterocolitis [17]. The use of the umbilical vein as a route for cardiac catheterization was first reported in 1961 [7]. Sapin et al. also reported using both the umbilical vein and umbilical artery for cardiac hemodynamics studies in 1963 [18]. Since the 1970s, more cardiac interventions that use the umbilical vein have been reported, including BAS, temporary pacemaker implantation, and the reinfusion route for venovenous extracorporeal membrane oxygenation (VV-ECMO) [19,20,21]. Simpson et al. reported using the umbilical vein route for pulmonary balloon valvuloplasty [22]. The umbilical artery has also been reported as a route for balloon dilatation of aortic coarctation and balloon aortic valvuloplasty [23,24]. Transcatheter PDA occlusion in ELBW infants became technically feasible following the development of a new delivery sheath and device [25]. Recent data show a tendency toward early improvement in the pulmonary score of a transcatheter occlusion group of infants compared with the surgical ligation group [26]. Previously, our group successfully used the umbilical vein for transcatheter closure of the PDA in symptomatic premature infants weighing more than 478 g [8]. The results implied the possibility of early prophylactic closure of the PDA in very small babies.

The DV is one of three physiological shunts of fetal circulation. The ductus reaches the IVC at the hepatic vein confluence with a steep angle (48° on average) [27]. After birth, functional closure of the ductus venosus begins when the ductus venosus blood pressure falls, and true obliteration is completed in 15–20 days [28,29]. In this case series, we found that early identification of the patency of the ductus venous to the IVC was important. According to our data, we had two patients for which cannulation failed due to ductus venous closure at days 3 and 4. In our report, the overall failure rate due to ductus venous closure was 12.5% (2/16). It was reported that bedside targeted ultrasonography performed by a physician can help in identifying mispositioned catheters [30,31] and that ultrasound-guided umbilical catheter insertion could save 64 min on average [32]. The DV size was, on average, 0.7 mm at 18 weeks GA and 1.7 mm at 40 weeks GA [27]. The umbilical vein diameter in a full-term baby was nearly the same as the outer diameter of a 4 Fr. introducer sheath (1.78 mm in the Merit sheath). However, the ductus venosus is a thin and soft vessel. It was reported that for a 10 Fr. catheter inserted into an umbilical vein for ECMO use in a GA 39-week infant with a BW of 3.3 kg, ECMO flow approached 250 mL/min and could be regarded as safe [21]. In our report, we inserted a 11 Fr. double-lumen catheter into a GA 32 + 6-week infant with a BW of 2356 g for 25 days for use in CVVH.

**Figure 3:**
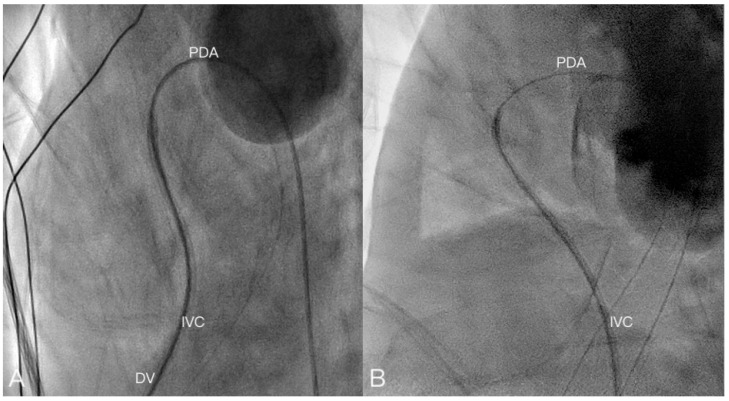
(**A**) Lateral view of transcatheter PDA occluder insertion through the UV route. The sheath passes through the angle between the DV and IVC until reaching the RVOT. The wire passes through the PDA until reaching the descending Ao (Case 10). (**B**) Lateral view of transcatheter PDA occluder insertion through the FV route. The sheath passes through the IVC straight to the RVOT, and the wire passes through the PDA until reaching the Ao.

**Figure 4:**
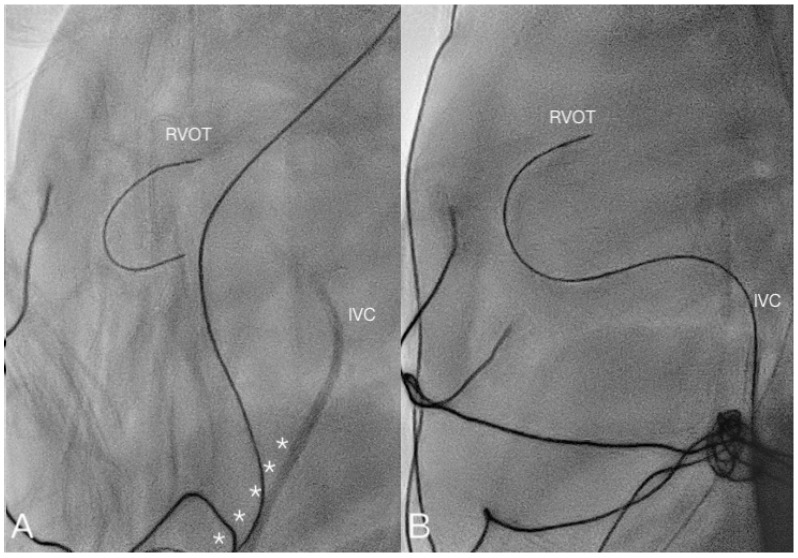
(**A**) Lateral view of transcatheter valvotomy through the UV route (Case 16). The curvature from the DV to IVC weakened the force to pass through the pulmonary valve (asterisk). (**B**) Lateral view of transcatheter valvotomy through the FV route.

In this study, we report our experience of using the umbilical vein as a route for intervention in 16 newborn cases representing 17 events. The smallest patient weighed 478 g and, to the best of our knowledge, is the smallest reported case. We performed PDA occlusion, balloon atrial septostomy, balloon pulmonary valvuloplasty, and pulmonary valve perforation smoothly via an umbilical vein route. The umbilical vein route was especially suitable for BAS. The ductus venosus continued on a straight path to the foramen ovale in early pregnancy [27]. The catheter could easily pass through the foramen ovale. It has been observed that a catheter encounters more difficultly in entering a right ventricle compared to the femoral vein route [6]. In eight cases, PDA occlusion was conducted. It was probably due to the help of the soft coronary guidewire that the guidewire could easily pass through the PDA and achieve occlusion (Figure 3, Supplementary Video S2). However, in one case, PV perforation was conducted. During the procedure, we initially used the guidewire approach for the RVOT. However, when we attempted to advance the guiding catheter and stiff wire to perforate the pulmonary valve, the force did not allow for the tip of the wire to be fully passed through the pulmonary valve. This may have been caused by the curvature between the ductus venosus and IVC. The curvature weakened the force, preventing the wire from passing through the pulmonary valve (Figure 4). In this report, the cardiac catheterization success rate is 73.3%. Intervention cardiac catheterization in premature infants, especially in extremely low birth weight (ELBW) infants, is more challenging because these patients are more fragile, and higher complication rates related to this procedure have been reported [22,25]. Postnatal age is related to the success rate of umbilical vein catheterization, and it was reported that the ductus venosus may be patent for cardiac catheterization in the first week of life [19,33]. The successful catheterization rate was higher when carried out within 48 h of life, but still with a 25% failure rate [13]. On the other hand, Ranjit et al. reported their experience in percutaneous cardiac intervention in infants weighing less than 1000 g. They reported that in two infants with pulmonary atresia, pulmonary valve perforations were successfully performed [34]. The same study group also demonstrated a short procedural time and fluoroscopic exposure time in transcatheter PDA occlusion among infants weighing less than 1000 g, contributing to the growing evidence of the feasibility of transcatheter cardiac intervention in these small infants [35]. The umbilical vein route may not be suitable for PV perforation in these patients, although the umbilical vein route may be used in PDA occlusion, BAS, and pulmonary valve balloon valvuloplasty.

Reported complications of using the umbilical vein as a catheterization route are infection of the umbilical stump, portal vein thrombosis, arterial thrombosis, arrhythmia, and intraperitoneal bleeding [6,33]. In this study, intraabdominal hemorrhage was noted for only one patient after this procedure, and it subsided after blood transfusion (Case 4).

The limitations of this study are the relatively small sample size and the use of a single center. However, we have constructed a program that is currently applicable and available for use in the umbilical vein approach for neonatal intervention. Further data from more applications of umbilical vein access in cardiac catheterization are needed to establish the safety of this procedure.

## 5. Conclusions

In this report, we presented our experience of neonatal intervention, especially in ELBW infants, via the umbilical vein. We have previously used the umbilical vein as a route for PDA occlusion, balloon pulmonary valvuloplasty, and balloon atrial septostomy. However, the umbilical vein may not be suitable in the perforation of pulmonary valves. Chest radiography and ultrasound may be used to check ductus venosus patency prior to the procedure. Cardiac catheterization and the placement of a large-sized catheter through the umbilical vein in a small infant may represent a safe and time-saving procedure, but more investigations are need to confirm this.

## Figures and Tables

**Table 1:** Baseline characteristics.

Case No.	Birth GA (Weeks)	Birth BW (g)	Sex	Age (Days)	BW at Procedure Day (g)	Diagnosis	Sheath Size (Fr.)
1	24 + 6	660	F	2	660	PDA with PH	4
2	23	568	M	12	509	PDA with PH	4
3	22 + 4	462	M	3	478	PDA with PH	4
4 *	32 + 6	2360	M	9	2356	PDA/renal hypoplasia	4/11
5	23 + 4	588	F	27	551	PDA	4
6	35 + 2	1675	M	3	1892	AKI	8
7	38 + 6	3705	M	1	3685	TGA	4
8	29 + 2	1442	F	7	1562	PDA	4
9	25 + 6	668	M	2	668	PDA	4
10	34 + 1	1330	M	6	1397	PDA with PH	6
11	39	3555	M	5	3444	Severe valvular PS	5
12	37 + 5	2392	F	3	2448	TGA	4
13	37 + 5	2500	M	1	2634	TGA	3
14	37 + 6	2576	F	7	2542	Severe valvular PS	6
15	39 + 2	2728	M	5	2812	Severe valvular PS	6
16	38 + 5	2692	M	5	2770	PAIVS	5

* Case 4 accounts for two intervention events (PDA closure and CVVH). Abbreviations: PS: pulmonary stenosis; PAIVS: pulmonary atresia with intact ventricular septum; TGA: transposition of the great arteries; PDA: patent ductus arteriosus; PH: pulmonary hemorrhage; AKI: acute kidney injury; PV: pulmonary valve; BAS: balloon atrial septostomy; CVVH: continuous venovenous hemofiltration.

**Table 2:** Intervention details.

Case No.	Event No.	Procedure	Sheath Size (Fr)	UV Cannulation	Device	Fluoroscopy Time (min s)	Procedure Time (min)	UV Cannulation Complications	Intervention Outcome
1	1	PDA occluder	4	Success	ADOIIAS 5-4	9 m 22 s	23		Expired due to sepsis post CoA operation at day 163
2	2	PDA occluder	4	Success	ADOIIAS 5-4	15 m 6 s	33		Discharge
3	3	PDA occluder	4	Success	ADOIIAS 3-2	10 m 22 s	40		Expired due to systemic fungal infection
4	4	PDA occluder	4	Success	ADOIIAS 5-4	19 m 54 s	57	Intraabdominal hemorrhage	Expired due to CVVH complication
	5	CVVH	11	Success					
5	6	PDA occluder	4	Failure (DV closed)	-	4 m 20 s	29		Shift to surgical ligation due to failure to venous cannulation; discharged
6	7	CVVH	8	Success					Discharged
7	8	BAS	4	Success	4 Fr. Bermann	5 m 50 s	59		Discharged after ASO operation
8	9	PDA occluder	4	Success	ADOIIAS 5-6	7 m 52 s	28		Expired due to ARPKD with renal failure at day 70
9	10	PDA occluder	4	Success	ADOIIAS 4-2	13 m 36 s	122		Bradycardia after device deployment then device retrieved; expired due to pulmonary hemorrhage 2 days later
10	11	PDA occluder	6	Success	AVPII 8-7	39 m 5 s	84		Discharged
11	12	Balloon valvuloplasty	5	Success	Mustang Balloon 8 mm × 20 mm	25 m 39 s	101		Discharged
12	13	BAS	4	Success	4 Fr. Bermann	13 m 29 s	34		Discharged after ASO operation
13	14	BAS	3	Failure (DV closed)	-	18 m 41 s	77		Shift to femoral vein for BAS, discharged after ASO operation
14	15	Balloon valvuloplasty	6	Success	Sterling Balloon 7 mm × 20 mm	23 m	40		Discharged
15	16	Balloon valvuloplasty	6	Success	Sterling Balloon 7 mm × 20 mm	19 m	56		Discharged
16	17	PV perforation	5	Success	-	50 m 18 s	145		PV perforation failure; femoral cannulation failure; shift to surgical pulmonary valvotomy and discharged

The device size is expressed in waist diameter × length (millimeter). Abbreviations: AKI: acute kidney injury; ARPKD: autosomal recessive polycystic kidney disease; ASO: arterial switch operation; ADOIIAS: Amplatzer Duct Occluder II Additional Size; AVPII: Amplatzer Vascular Plug II; BAS: balloon atrial septostomy; CoA: coarctation of aorta; CVVH: continuous venovenous hemofiltration; DAP: dose-area product; DV: ductus venous; PAIVS: pulmonary atresia with intact ventricular septum; PDA: patent ductus arteriosus; PH: pulmonary hemorrhage; PS: pulmonary stenosis; PV: pulmonary valve; TGA: transposition of the great arteries; UV: umbilical vein.^.^

## Data Availability

The datasets used during the current study are available from the corresponding author on reasonable request.

## Supplementary Materials

The following are available online at https://www.mdpi.com/article/10.3390/children8111017/s1, Video S1: Angiogram showing closure of DV (Case 5). Video S2: The 0.014 inch soft coronary wire can guide the catheter into PDA.
